# Doxorubicin Induces Inflammatory Modulation and Metabolic Dysregulation in Diabetic Skeletal Muscle

**DOI:** 10.3389/fphys.2016.00323

**Published:** 2016-07-27

**Authors:** Rashmi Supriya, Bjorn T. Tam, Xiao M. Pei, Christopher W. Lai, Lawrence W. Chan, Benjamin Y. Yung, Parco M. Siu

**Affiliations:** Department of Health Technology and Informatics, Faculty of Health and Social Sciences, The Hong Kong Polytechnic UniversityHong Kong, China

**Keywords:** type 2 diabetes mellitus, cancer chemotherapy, myotoxicity, anaerobic glycolysis, pro-inflammation, anti-inflammation

## Abstract

Anti-cancer agent doxorubicin (DOX) has been demonstrated to worsen insulin signaling, engender muscle atrophy, trigger pro-inflammation, and induce a shift to anaerobic glycolytic metabolism in skeletal muscle. The myotoxicity of DOX in diabetic skeletal muscle remains largely unclear. This study examined the effects of DOX on insulin signaling, muscle atrophy, pro-/anti-inflammatory microenvironment, and glycolysis metabolic regulation in skeletal muscle of db/db diabetic and db/+ non-diabetic mice. Non-diabetic db/+ mice and diabetic db/db mice were randomly assigned to the following groups: db/+CON, db/+DOX, db/dbCON, and db/dbDOX. Mice in db/+DOX and db/dbDOX groups were intraperitoneally injected with DOX at a dose of 15 mg per kg body weight whereas mice in db/+CON and db/dbCON groups were injected with the same volume of saline instead of DOX. Gastrocnemius was immediately harvested, weighed, washed with cold phosphate buffered saline, frozen in liquid nitrogen, and stored at −80°C for later analysis. The effects of DOX on diabetic muscle were neither seen in insulin signaling markers (Glut4, pIRS1Ser^636∕639^, and pAktSer^473^) nor muscle atrophy markers (muscle mass, MuRF1 and MAFbx). However, DOX exposure resulted in enhancement of pro-inflammatory favoring microenvironment (as indicated by TNF-α, HIFα and pNFκBp65) accompanied by diminution of anti-inflammatory favoring microenvironment (as indicated by IL15, PGC1α and pAMPKβ1Ser108). Metabolism of diabetic muscle was shifted to anaerobic glycolysis after DOX exposure as demonstrated by our analyses of PDK4, LDH and pACCSer^79^. Our results demonstrated that there might be a link between inflammatory modulation and the dysregulation of aerobic glycolytic metabolism in DOX-injured diabetic skeletal muscle. These findings help to understand the pathogenesis of DOX-induced myotoxicity in diabetic muscle.

## Introduction

Doxorubicin (DOX) is an effective chemotherapeutic drug for treating various types of cancer (Cortés-Funes and Coronado, 2007; Tacar et al., 2013). Nonetheless, the side effects of DOX on cardiomyocytes leading to life-threatening cardiomyopathy have limited its clinical use (Swain et al., 2003). Indeed, the adverse side effects of DOX have also been documented in the brain (Aluise et al., 2010), liver (Gokcimen et al., 2007), kidney (Wapstra et al., 1999), lung (Lim et al., 2010), blood vessels (Murata et al., 2001), and skeletal muscle (Smuder et al., 2011; Hayward et al., 2013). DOX is a potent drug for chemotherapy and it reflects a common picture of the chemotherapeutic drugs used for treating cancer (Shaikh and Shih, 2012). Generation of reactive oxygen species (ROS) and apoptosis-inducing characteristics are the common features shared by the anticancer drugs (Minotti et al., 2004; Nitiss, 2009; Gilliam and St. Clair, 2011). DOX has been demonstrated to adversely affect the muscle quality and muscle mass (Falkenberg et al., 2001; Gilliam and St. Clair, 2011; Smuder et al., 2011). DOX treatment was demonstrated to increase calcium flux from isolated sarcoplasmic reticulum vesicles in skeletal muscle cells (van Norren et al., 2009). DOX has also been shown to reduce calcium sensitivity by interfering with actin-myosin interaction in skeletal muscle (Hydock et al., 2011). Other adverse physiological consequences of DOX in skeletal muscle include muscle atrophy, muscle cell death, muscle mass reduction, and decrease in maximal twitch force (Falkenberg et al., 2001; Arthur et al., 2008; Gilliam and St. Clair, 2011; Smuder et al., 2011). DOX is comprised of a quinone moiety in its chemical structure, which is responsible for causing oxidative stress by interacting with molecular oxygen in skeletal muscle fibers (Chen et al., 2007). The resultant elevated oxidative stress disrupts several cellular mechanisms involving calpain and caspase-3 protease (Gilliam and St. Clair, 2011), AMP-activated protein kinase (AMPK) (Irrcher et al., 2008), and insulin receptor substrate 1 (IRS1) (Martins et al., 2012), in which DOX impairs the processes of apoptosis, autophagy, insulin signaling, and inflammatory pathways.

Inflammation and dysregulated glycolytic metabolism are reported in type 2 diabetes mellitus (Simoneau and Kelley, 1997; Pickup, 2004). Oxidative stress, inflammatory modulation and dysregulated glycolytic metabolism have also been reported in the DOX-exposed skeletal muscle (van Etten et al., 1998; Hardin et al., 2008; van Norren et al., 2009). Diabetes in skeletal muscle weakens the tricarboxylic acid (TCA) cycle (Ritov et al., 2010) and releases enormous amount of ROS by downregulating peroxisome proliferator-activated receptor gamma coactivator 1-alpha (PGC1α) (Mootha et al., 2003; Sparks et al., 2005), which is one of the important mediators in the DOX-induced skeletal muscle myopathy (Min et al., 2015). ROS-PGC1α signaling is a key pathway in the regulation of mitochondrial function in skeletal muscle and they are inversely related to each other (Liao, 2012). The augmented oxidative stress induced by DOX in diabetic skeletal muscle might cause metabolic dysregulation by inhibiting oxidative phosphorylation and upregulating lactate dehydrogenase (LDH) (Tannahill et al., 2013). The upregulated LDH leads to the activation of hypoxia-induced factor 1α (HIF1α) (Tannahill et al., 2013) and nuclear factor kappa B (NFκB) (Remels et al., 2014), which are transcription factors and regulatory molecules that favor toward pro-inflammatory microenvironment and trigger the secretion of pro-inflammatory cytokines [e.g., tumor necrosis factor alpha (TNFα) (Hotamisligil and Spiegelman, 1994) and Interleukin 6 (IL6) (Franckhauser et al., 2008)]. On the other hand, DOX has also been demonstrated to induce oxidative stress in skeletal muscle without altering LDH (van Norren et al., 2009) but to cause insulin resistance in muscle by downregulating IRS-1, glucose transporter type 4 (GLUT4), AMPK and glycogen synthase kinase 3 beta (GSK3β) (Hayward et al., 2013). While AMPK downregulation is suggested to be involved in insulin resistance development in skeletal muscle treated with DOX (Viollet et al., 2009), AMPK also acts as a metabolic master switch that phosphorylates target proteins in fatty acid oxidation metabolic pathways in skeletal muscle (Viollet et al., 2009). Besides, AMPK acts as a regulatory molecule favoring the anti-inflammatory microenvironment leading to the shift toward oxidative metabolism (Sag et al., 2008), which reduces glycolytic rate by activating PGC1α that ameliorates inflammatory cytokines/myokines such as TNFα and IL6 (Ostrowski et al., 1999; Steinberg et al., 2006) and enhances anti-inflammatory cytokines/myokines such as IL15 and IL10 (Wang et al., 2014; Crane et al., 2015).

Approximately 8–18% of patients with cancer are diabetic (Richardson and Pollack, 2005). Also, patients with diabetes are at a higher risk of developing cancers in breast, pancreatic, liver, kidney, endometrial, and colon (Richardson and Pollack, 2005). Though the exact links between diabetes and cancer are not known, the exposure to hyperglycemia, elevated insulin, and growth-promoting IGF-1 have been postulated to be the possible reasons explaining the increased incidence of cancers in diabetic patients (Grimberg, 2003). Nevertheless, patients with diabetes and cancer need to be particularly considered for undergoing chemotherapy due to the detrimental side effects of the chemotherapeutic drugs (Psarakis, 2006). In regard to DOX myotoxicity, DOX has been demonstrated to worsen insulin signaling, engender muscle atrophy, disseminate pro-inflammation, and induce an oxidative-to-glycolytic metabolic shift in normal skeletal muscle (Braun et al., 2014; Fabris and MacLean, 2015; de Lima Junior et al., 2016). However, the effects of DOX on skeletal muscle of diabetic individuals are largely unclear and the responsible molecular mechanisms in diabetic muscle remain to be elucidated. This study aimed to examine the effects of DOX on insulin signaling, muscle atrophy, pro-/anti-inflammatory microenvironment, and glycolysis metabolic regulation in skeletal muscle of diabetic animals.

## Methods

### Animal

Male 14- to 18-week-old db/db mice were obtained from the Laboratory Animal Services Centre of The Chinese University of Hong Kong. The db/db mouse is a well-established leptin receptor-deficient animal model (homozygous allelic deficient for the leptin receptor gene) that mimics the disease phenotype of human type 2 diabetes mellitus. Non-diabetic db/+ mice (heterozygous allele deficient for the leptin receptor gene) of the same genetic background as db/db mice were used as control group. Mice were housed in a humidity- and temperature-controlled environment and were exposed to a 12:12-h light:dark cycle in the Centralized Animal Facilities of The Hong Kong Polytechnic University. Mice were allowed to have access to standard animal diet and water ad libitum. Animal ethics approval was obtained from the Animal Ethics Sub-committee of The Hong Kong Polytechnic University.

### Experimental protocol

Non-diabetic db/+ mice and diabetic db/db mice were randomly assigned to the following groups: db/+CON, db/+DOX, db/dbCON, and db/dbDOX (*n* = 5 per group). The diabetic status of our examined db/db mice was confirmed by the measurements of fasting blood glucose level (db/db mice vs. db/+ mice: 27.1 ± 1.0 mmol/L vs. 7.8 ± 0.5 mmol/L) and HbA1c level (db/db mice vs. db/+ mice: 7.0 ± 0.4% vs. 5.8 ± 0.1%). Mice in db/+DOX and db/dbDOX groups were intraperitoneally injected at one time point with DOX (Pharmacia and Upjohn SpA, Milan, Italy) at a dose of 15 mg per kg body weight whereas mice in db/+CON and db/dbCON groups were injected with the same volume of saline instead of DOX (Yu et al., 2014; Pei et al., 2015). Five days after the DOX administration, the gastrocnemius was immediately harvested, weighed, washed with cold phosphate buffered saline (PBS), frozen in liquid nitrogen, and stored at −80°C for later analysis (Yu et al., 2014).

### Protein fraction preparation

Protein fractions were extracted from muscle homogenates as previously described (Yu et al., 2014; Pei et al., 2015) Forty mg of sample tissue were minced and homogenized in ice-cold lysis buffer (10 mmol/L NaCl, 1.5 mmol/L MgCl_2_, 20 mmol/L HEPES, pH 7.40, 20% glycerol, 0.1% Triton X-100, and 1 mM dithiothreitol). Homogenates were subject to centrifugation at 875 × g for 5 min at 4°C. The supernatant was obtained and subject to further centrifugation at 3500 × g for 5 min at 4°C in which these procedures were repeated thrice. Finally, the supernatant was collected as the cytoplasmic protein fraction. Then, protease inhibitor cocktail (P8340, Sigma-Aldrich) was added to the cytoplasmic protein fraction. Protein concentration was quantified in triplicates by Bradford assay (Coomassie Protein Assay, Pierce) with bovine serum albumin used as the standard.

### Western blot analysis

The protein abundances of insulin signaling markers (IRS-1Ser^636∕639^, AktSer^473^, GLUT4), muscle atrophy markers (MuRF1 and MAFbx), markers for pro-inflammatory favoring microenvironment (TNFα, IL6, HIF1α, pNFkBp65), markers for anti-inflammatory favoring microenvironment (IL10, IL15, pAMPKβ1Ser^108^, PGC1α) and metabolic regulators (PDK4, pACCSer^79^, LDH) were evaluated by Western blotting. Forty micrograms of protein were denatured at 95°C for 5 min in Laemmli buffer with 5% β-mercaptoethanol. The protein samples were subject to gel electrophoresis on 10% SDS-PAGE gel. Resolved proteins were then transferred to polyvinylidene difluoride (PVDF) membranes (Immobilon-P, Millipore) by using the Bio-Rad Mini-Protein II system. The membrane was then blocked with 5% skimmed milk powder in PBS/0.1% Tween-20 (PBST) followed by incubation with respective primary antibodies overnight at 4°C. The primary anti-bodies used for probing various markers, their sources and dilutions used are listed in the Table 1. Membranes were incubated with appropriate secondary antibodies (i.e., anti-mouse, anti-rabbit or anti-goat IgG horseradish peroxidase-conjugated antibodies; Cell Signaling, 1:4000) after washing. The immunoreactivity was determined using the ECL chemiluminescence reaction kit (Perkin Elmer) and the images were captured by Chemi Doc (Bio-Rad camera, USA). GAPDH was used as the internal loading control. The arbitrary units of the blot signal are presented as net intensity x band area, normalized to the signal of GAPDH or the respective total protein for phosphorylation status.

**Table 1:** **List of antibodies used**.

**Antibody**	**Dilution factor**	**Source**
Anti-phospho-IRS1 (Ser636/639) rabbit polyclonal	1:1000	2388, Cell signaling technology
Anti-phospho-Akt (Ser473) rabbit polyclonal	1:1000	9271, Cell signaling technology
Anti-Akt rabbit polyclonal	1:1000	9272, Cell signaling technology
Anti GLUT4 rabbit polyclonal	1:500	07-1404, Millipore
Anti-MuRF1 rabbit polyclonal	1:500	32920, Santa Cruz
Anti-MAFbx rabbit polyclonal	1:500	33782, Santa Cruz
Anti-phospho-AMPKβ1(Ser108) rabbit polyclonal	1:1000	4181, Cell signaling technology
Anti PGC1α rabbit polyclonal	1:500	13067, Santa Cruz
Anti-IL10 goat polyclonal	1:1000	365858, Santa Cruz
Anti-IL15 goat polyclonal	1:1000	1296, Santa Cruz
Anti-phospho-NFkβ p65 rabbit monoclonal	1:1000	3033, Cell signaling technology
Anti-HIF1α rabbit polyclonal	1:500	10790, Santa Cruz
Anti-TNFα goat polyclonal	1:1000	52746, Santa Cruz
Anti-IL6 goat polyclonal	1:1000	1265, Santa Cruz
Anti-PDK4 (Thr410/403) goat polyclonal	1:1000	14495, Santa Cruz
Anti-phospho-ACC (Ser79) rabbit polyclonal	1:1000	3661, Cell signaling technology
Anti-LDH rabbit polyclonal	1:500	33781, Santa Cruz

### Lactate dehydrogenase (LDH) activity

Lactate dehydrogenase (LDH) activity was determined by a commercially available kit (ab102526, Lactate Dehydrogenase Activity Colorimetric Assay Kit, Abcam). The assay was performed according to the manufacturer's instruction. In brief, LDH converts pyruvate into lactate that reduces the developer to a colored product with absorbance at 450 nm measured by a microplate reader (Infinite F200, Tecan, Switzerland).

### Data analyses

Statistical analyses were performed by using the SPSS 21.0 software package (IBM, Chicago, IL, USA). Normality tests were performed to examine data distribution. All data were expressed as mean ± standard error of the mean (SEM). Two-way ANOVA was used to examine the interaction and main effects of the two experimental factors (i.e., diabetes and DOX) and subsequent Tukey's HSD post-hoc test was used to examine the simple effect if a significant interaction effect was found. Statistical significance was set at *p* < 0.05. Of note, the adopted sample size in the present study resulted in a statistical power of 80% or above for the assessments including muscle mass, IRS1, MAFbx, IL6, IL10, IL15, AMPK, PGC1α, HIF1α, and LDH activity.

## Results

### No exacerbating effect of DOX exposure on insulin signaling in diabetic muscle

**Figure 1:**
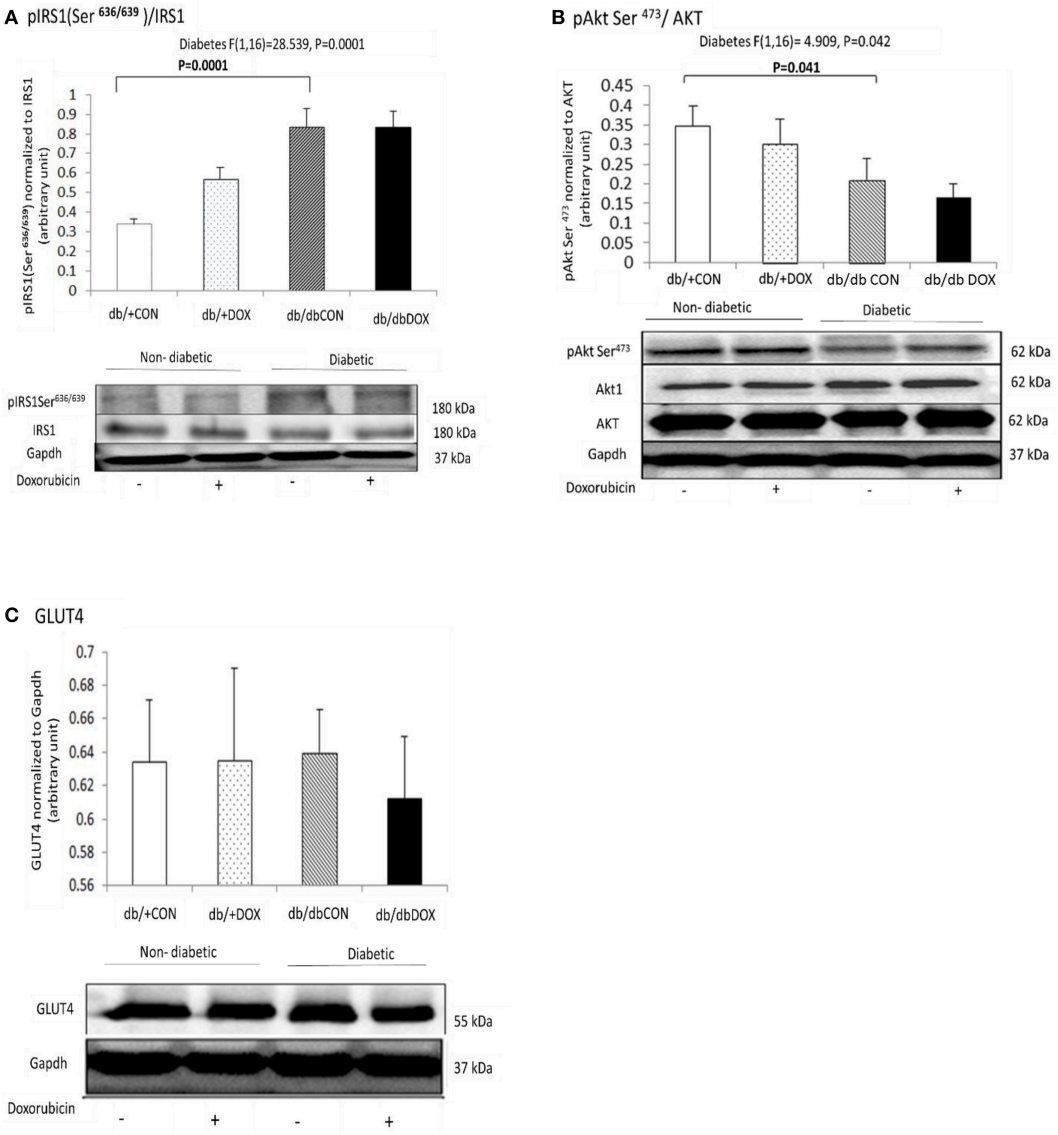
**Non-diabetic and diabetic skeletal muscle insulin signaling after DOX exposure examined by phosphorylation statuses of IRS1(Ser636/639) (A), Akt (Ser473) (B), and GLUT4 (C) are shown**. Data are expressed as mean ± SEM.

The abundance of insulin signaling proteins including phosphor-IRS1Ser^636∕639^, phosphor-AKTSer^473^ and GLUT4 were measured in the gastrocnemius muscle. No interaction effect of diabetes and DOX was observed in all the three markers. We observed the main effect of diabetes for phosphor-IRS-1 (*P* = 0.0001) and the abundance of protein was significantly increased by 145% (*P* = 0.0001) in the gastrocnemius muscle of db/dbCON mice relative to that of db/+CON mice (Figure 1A). There was a main effect of diabetes for AKT (*P* = 0.042), and the protein abundance of AKT was significantly reduced by 61% (*P* = 0.041) in the muscle of db/dbCON mice relative to db/+CON mice (Figure 1B). We did not observe interaction effect of diabetes with DOX or significant main effect of diabetes in GLUT4 (Figure 1C).

### No exacerbating effect of DOX exposure on muscle atrophy in diabetic muscle

**Figure 2:**
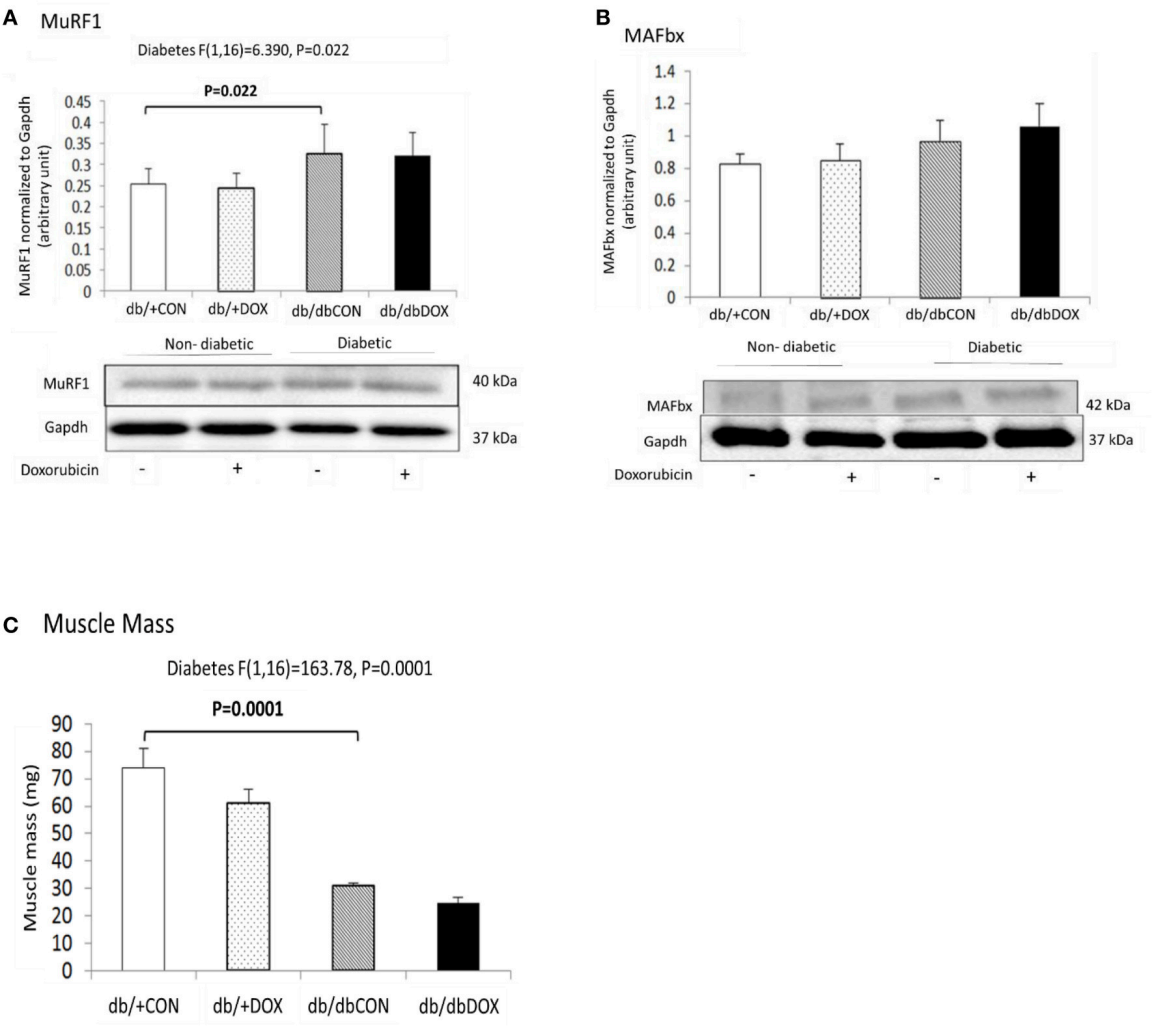
**Non-diabetic and diabetic skeletal muscle atrophy after DOX exposure examined by phosphorylation statuses of MuRF1 (A) and MAFbx (B) muscle mass (mg) (C) are shown**. Data are expressed as mean ± SEM.

The abundance of muscle atrophy markers including MuRF1, MAFbx and muscle mass were measured in the gastrocnemius muscle. No interaction effect of diabetes with DOX was found for MuRF1. There was a significant main effect of diabetes for MuRF1 (*P* = 0.022) and a significant 48.28% (*P* = 0.022) increase in protein abundance of MuRF1 in muscle of db/dbCON mice relative to that of db/+CON mice (Figure 2A). We did not observe any significant interaction effect for MAFbx. Also, no significant main effect of diabetes was found in MAFbx (Figure 2B). No significant interaction effect was observed in the muscle mass. However, significant main effect of diabetes was observed in muscle mass reduction (*P* = 0.0001) and muscle mass was significantly decreased by 58% (*P* = 0.0001) in the gastrocnemius muscle of db/dbCON mice relative to that of db/+CON mice (Figure 2C).

### DOX caused anti-inflammatory microenvironment diminution and pro-inflammatory microenvironment augmentation in diabetic muscle

**Figure 3:**
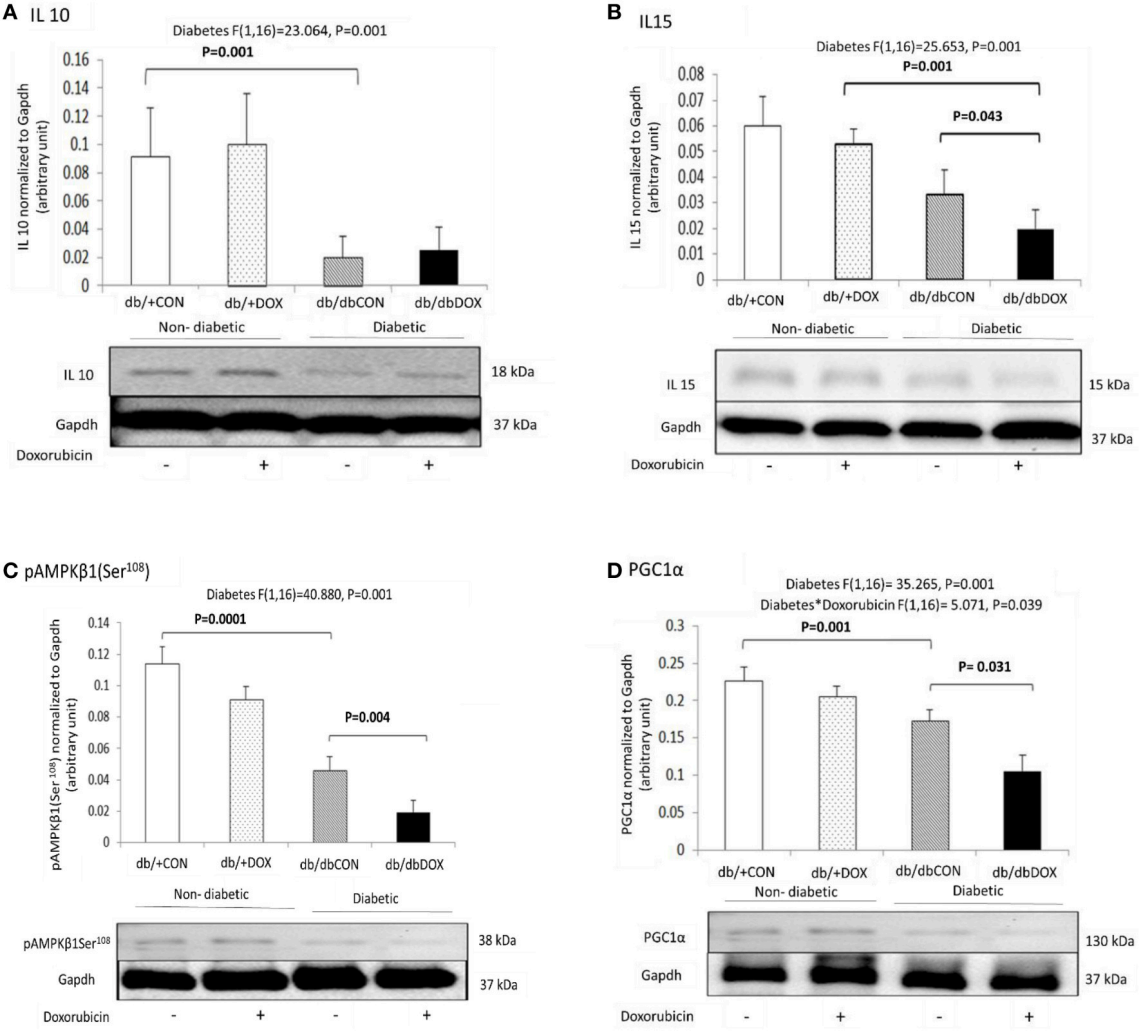
**Non-diabetic and diabetic skeletal muscle anti-inflammatory microenvironment after DOX exposure examined by phosphorylation of myokines: IL10 (A), IL15 (B) AMPKβ1Ser108 (key regulator) (C) and PGC1α (transcription factor) (D) are shown**. Data are expressed as mean ± SEM.

The muscle-specific cytokines, transcription factor and regulatory molecule that modulate anti-inflammatory microenvironment were examined. No interaction effect of diabetes with DOX was observed for IL10. Significant main effect of diabetes was observed in IL10 (*P* = 0.001) and its protein abundance significantly decreased by 78% (*P* = 0.001) in the muscle of db/dbCON mice relative to that of db/+CON mice (Figure 3A). No interaction effect of diabetes with DOX was observed for IL15. Significant main effect of diabetes was found in IL15 (*P* = 0.001). The protein abundance of IL15 was significantly decreased by 51% (*P* = 0.001) in muscle of db/dbDOX mice relative to the muscle of db/+DOX mice and it was also significantly decreased by 66% (*P* = 0.043) in the muscle of db/dbDOX mice relative to db/dbCON mice (Figure 3B). Similarly, no interaction effect of diabetes and DOX was observed in AMPK. Significant main effect of diabetes was found in AMPK (*P* = 0.0001). There was a significant 62% (*P* = 0.0001) decrease in protein abundance of phosphor AMPKβ1Ser108 in muscle of db/dbCON mice relative to db/+CON mice and was also significantly decreased by 57% (*P* = 0.004) in db/dbDOX mice relative to that of db/dbCON mice (Figure 3C). Significant interaction effect of diabetes with DOX (*P* = 0.039) for PGC-1α was found. A significant 31% (*P* = 0.001) decrease in protein abundance of PGC-1α in the muscle of db/dbCON relative to the muscle of db/+CON mice and also a significant 36% (*P* = 0.031) decrease in the muscle of db/dbDOX relative to that of db/dbCON mice was observed (Figure 3D).

**Figure 4:**
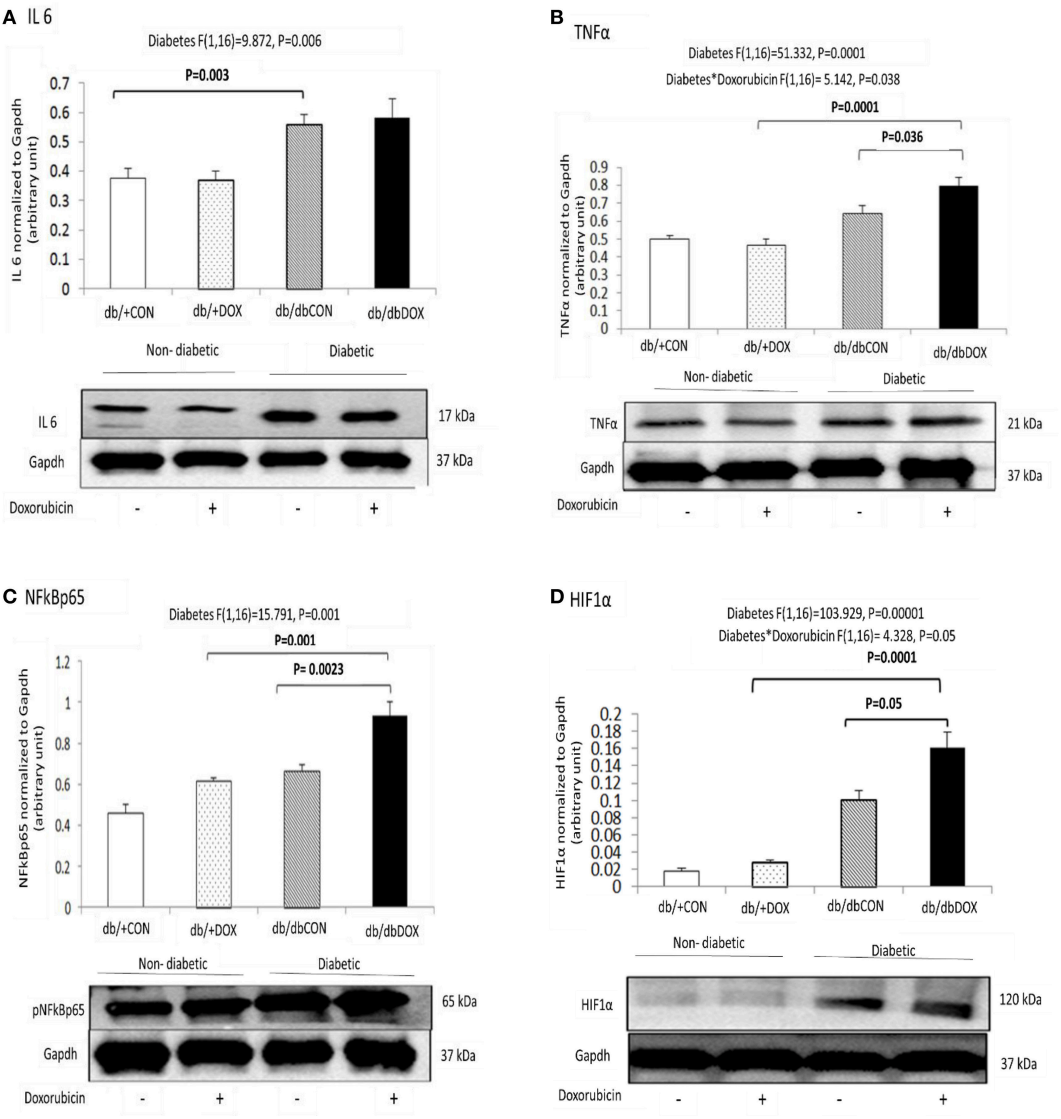
**Non-diabetic and diabetic skeletal muscle pro-inflammatory microenvironment after DOX exposure examined by phosphorylation statuses of myokines: IL6 (A) TNFα (B) pNFkBp65 (Key regulator) (C) and HIF1α (transcription factor) (D) are shown**. Data are expressed as mean ± SEM.

The muscle-specific cytokines, transcription factor and regulatory molecule that modulate pro-inflammatory favoring microenvironment were assessed. No interaction effect of diabetes with DOX was observed for IL6. Significant main effect of diabetes was found in IL6 (*P* = 0.006). The protein abundance of IL6 was significantly increased by 37% (*P* = 0.003) in muscle of db/dbCON mice relative to that of db/+CON mice (Figure 4A). Significant interaction effect of diabetes and DOX was observed for TNFα (*P* = 0.038). The protein abundance of TNFα was increased by 35% (*P* = 0.0001) in muscle of db/dbDOX mice relative to that of db/+DOX. TNFα was also increased by 13% (*P* = 0.036) in the muscle of db/dbDOX mice relative to db/dbCON mice (Figure 4B). No interaction effect of diabetes and DOX was observed for NFkB. Significant main effect of diabetes was observed for NFkB (*P* = 0.001). There was a significant 47% (*P* = 0.001) increase in phosphor-NFkB in db/dbDOX muscle relative to db/+DOX muscle and a significant 46% (*P* = 0.0023) increase in phosphor-NFkB in db/dbDOX muscle relative to db/dbCON muscle (Figure 4C). Significant interaction effect of diabetes with DOX was observed in HIF-1α (*P* = 0.05). The protein abundance of HIF-1α was increased by 635% (*P* = 0.0001) in muscle of db/dbDOX mice relative to db/+DOX mice. HIF-1α and increased by 47% (*P* = 0.05) in db/dbDOX mice relative to that of db/dbCON mice (Figure 4D).

### DOX caused a shift toward anaerobic glycolysis in diabetic muscle

**Figure 5:**
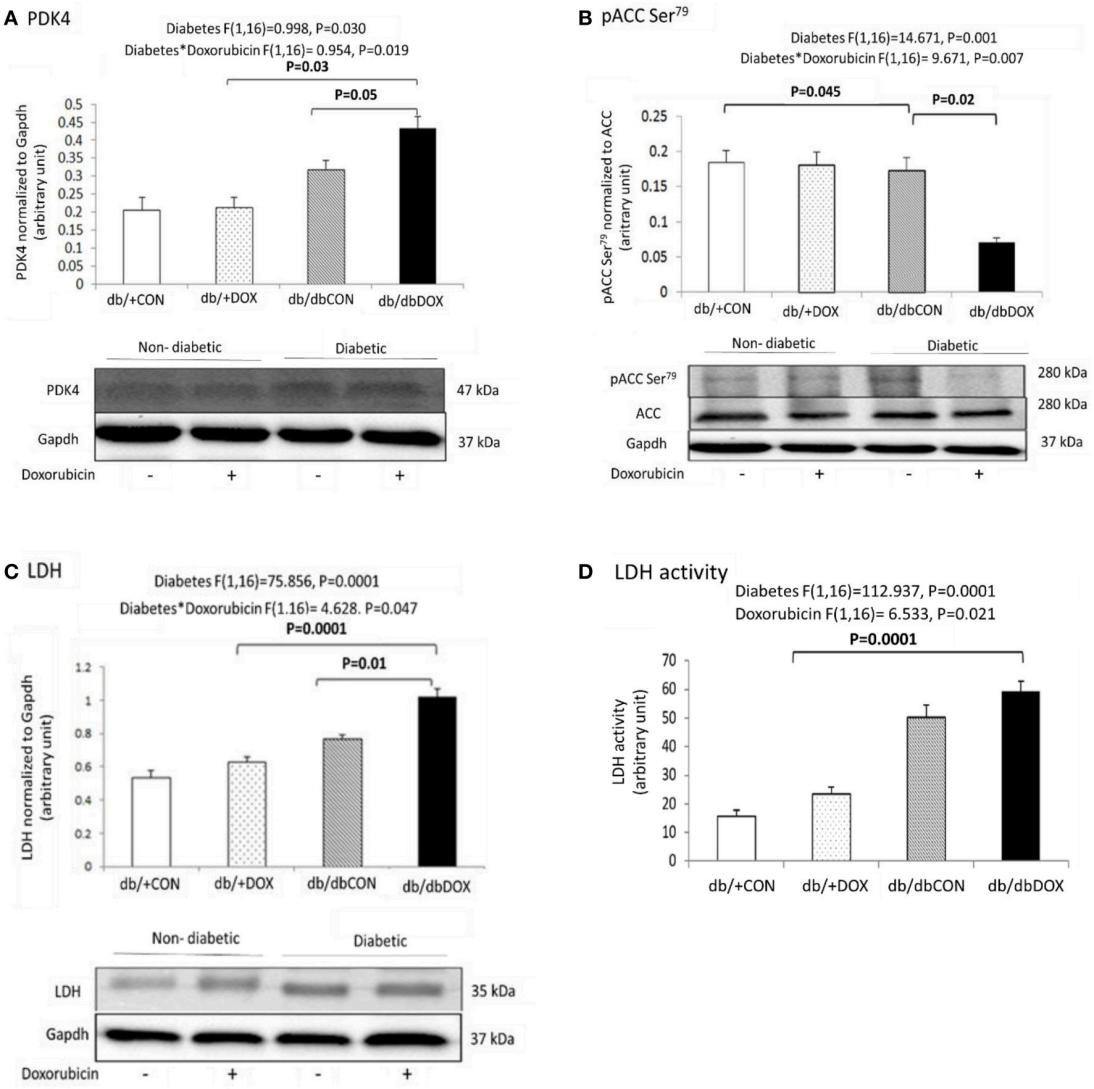
**Non-diabetic and diabetic skeletal muscle glycolytic mechanism after DOX exposure examined by phosphorylation statuses of PDK4 (A) and ACC (Ser79) (B) LDH protein (C) LDH activity (D) are shown**. Data are expressed as mean ± SEM.

Anaerobic glycolysis favoring metabolic regulators including pyruvate dehydrogenase lipoamide kinase isozyme 4 (PDK4), ACC, and LDH were examined. Significant interaction effect of diabetes with DOX was observed in PDK4 (*P* = 0.019). The protein abundance of PDK4 significantly increased by 94% (*P* = 0.03) in the muscle of db/dbDOX mice relative to the muscle of db/+DOX mice and significantly increased by 34% (*P* = 0.05) in the muscle of db/dbDOX mice relative to that of db/dbCON mice (Figure 5A). Significant interaction effect of diabetes with DOX was observed in ACC (*P* = 0.007). There was a significant 12% (*P* = 0.045) decrease in protein abundance of phosphor-ACC in the muscle of db/dbCON relative to that of db/+CON mice. Muscle of db/dbDOX mice showed a significant 68% (*P* = 0.02) decrease in protein abundance of phosphor-ACC relative to that of db/dbCON mice (Figure 5B). Significant interaction effect of diabetes and DOX was observed for both LDH protein abundance (*P* = 0.0001) and LDH activity (*P* = 0.047). There was a significant 47% (*P* = 0.0001) increase in the protein abundance of LDH in the muscle of db/dbDOX mice relative to db/+DOX mice and a 31% (*P* = 0.01) increase in LDH in the muscle of db/dbDOX mice relative to that of db/dbCON (Figure 5C). A significant 134% (*P* = 0.001) increase in LDH activity in muscle of db/dbDOX mice relative to that of db/+DOX mice was found (Figure 5D).

## Discussion

In this study, we demonstrated that DOX treatment in diabetic skeletal muscle did not exacerbate the impairment of insulin signaling and muscle atrophy as compared to non-diabetic skeletal muscle. However, DOX augmented pro-inflammatory microenvironment in diabetic muscle by upregulating transcription factor HIF1α, regulatory molecule pNFκBp65, and cytokine TNFα. DOX also altered anti-inflammatory microenvironment by downregulating transcription factor PGC1α, regulatory molecule pAMPKβ1Ser108 and muscle-specific myokine IL15. In addition, DOX induced dysregulated glycolytic metabolism in diabetic skeletal muscle by upregulating PDK4 and LDH and downregulating phosphorylation of acetyl-CoA carboxylase (pACCSer79).

Type 2 diabetes (T2D) is a progressive chronic disease, primarily characterized by functional decline of beta cell, worsening of insulin signaling, and muscle atrophy (Trostler et al., 1982; Cotter et al., 1993; Fonseca, 2009; Brannmark et al., 2013; de Lima Junior et al., 2016). DOX administration in non-diabetic skeletal muscle has been shown to cause insulin resistance and degradation of muscle quality (Hydock et al., 2011; Smuder et al., 2011; de Lima Junior et al., 2016). In the present study, our results are consistent with the previous findings of insulin signaling and muscle atrophy markers in diabetic muscle and DOX-treated muscle. However, our data indicated that there was no significant difference in insulin signaling and muscle atrophy markers in DOX-treated diabetic skeletal muscles when compared to diabetic skeletal muscles without DOX exposure. We interpreted that, in diabetic skeletal muscle, the diabetic characteristics (i.e., insulin signaling and muscle atrophy) were already worsened and hence the DOX exposure did not lead to a further impairment of insulin signaling and muscle atrophy markers. We did not observe significant difference in anti-inflammatory microenvironment (IL10, IL15, AMPK, and PGC1α), pro-inflammatory microenvironment (IL 6, TNF α, NFkB, and HIF1α) as well as in metabolic regulators (ACC, PDK4, and LDH) in skeletal muscles treated with DOX when compared to skeletal muscle without DOX exposure. We inferred that, DOX exposure may primarily affect the insulin sensitivity (de Lima Junior et al., 2016) and muscle atrophy (Chen et al., 2007) due to the accumulation of reactive oxygen species in the skeletal muscle (van Etten et al., 1998; Hardin et al., 2008; van Norren et al., 2009), and this is affected by the modulation of inflammatory microenvironment and metabolic dysregulation in the plasma (Haus et al., 2009). Nevertheless, our results suggested that DOX administration in diabetic skeletal muscle might cause an environment which is favorable toward aggregating pathogenesis of T2D as indicated by our observations of the inflammatory upsurge and metabolic dysregulation (Rhodes, 2005; Fonseca, 2009; Rocha et al., 2016).

### The link of diminution of anti-inflammatory microenvironment and dysregulated oxidative metabolism

AMPK, a regulatory molecule favoring anti-inflammatory microenvironment (O'Neill and Hardie, 2013), directly activates PGC1α (Jäger et al., 2007) and regulates IL15 (Crane et al., 2015). Therefore, downregulation of AMPK in diabetic skeletal muscles after DOX treatment leading to the reduction of PGC1α and IL15 might indicate the diminution of the anti-inflammatory microenvironment, as shown in our results. AMPK activation in skeletal muscle has been shown to result in muscle fiber shifting from glycolytic fibers to oxidative fibers in a PGC1α-dependent manner (Garcia-Roves et al., 2008). AMPK was also demonstrated to stimulate fatty acid oxidation and reduce the activity of acetyl-coenzyme A (CoA) carboxylase, which is an enzyme carrying out the conversion of acetyl CoA to malonyl CoA (Castle et al., 2009) that directs fatty acid oxidation in skeletal muscle (Abu-Elheiga et al., 2001). Since the phosphorylation of ACC was significantly downregulated in our examined muscles, we suspected that DOX decreased fatty acid oxidative metabolism in diabetic skeletal muscle. It has been reported that oxidative phosphorylation (OXPHOS) genes are strongly correlated to PGC1α in diabetic skeletal muscle based on DNA microarray analysis (Mootha et al., 2003); also, it has been reported that DOX treatment in the heart inhibited oxidative phosphorylation (Abdel-aleem et al., 1997) and reduced phosphorylation of ACC in white adipose tissue (Biondo et al., 2016). Consistently, we demonstrated in our examined muscle, the decreases in AMPK and PGC1α, which induced glycolytic shift as indicated by the downregulation of pACCSer79. Overall, our findings suggest that there might be a link between anti-inflammatory microenvironment and dysregulated metabolism in the DOX-induced myotoxicity in diabetic skeletal muscle. Further research is needed to fully dissect the regulatory mechanisms of the link the inflammatory microenvironment and metabolism dysregulation in DOX-injured diabetic skeletal muscle.

### The link of augmentation of pro-inflammatory microenvironment and anaerobic glycolytic metabolism

NFκB, a regulatory molecule favoring pro-inflammatory microenvironment (Eisele et al., 2013) has been shown to be activated by ROS (Dodd et al., 2010) and TNFα (Remels et al., 2014) in skeletal muscle. Additionally, classical activation of NFκB was demonstrated to augment muscle glycolytic metabolism in a HIF1α-dependent manner (Remels et al., 2014). Our results exhibited that the upregulation of TNFα activated NFκB and augmented glycolytic metabolism in HIF1α-dependent manner. Notably, DOX has been reported to induce cardiotoxicity by decreasing conversion of acetyl-CoA to malonyl CoA and the decreased rate of conversion inhibited fatty acid oxidation and ATP generation (Peluso et al., 2000). Consistently, an anti-diabetic drug (metformin) has been shown to prevent cardiotoxicity induced by DOX by increasing fatty acid oxidation and preventing energy starvation (Ashour et al., 2012). Metformin in combination with DOX has been demonstrated to inhibit the inflammatory pathway by inhibiting NFκB (a pro-inflammatory regulatory molecule) in mammalian cancer cell line (Hirsch et al., 2013). We suspected that if metformin suppressed the effect of DOX toxicity by enhancing phosphorylation of ACC and decreasing NFκB, then DOX induction along with diabetes might reduce ACC phosphorylation and aggravate inflammation by triggering NFκB according to the present findings. Since NFκB augmented muscle glycolytic metabolism in a HIF1α-dependent manner (Remels et al., 2014), HIF1α upregulation might be an indicator of an increase in glycolytic metabolism, as shown in our data. Our observed increase in TNFα suggested that NFκB was increased in a PGC1α-dependent fashion as it has been demonstrated that TNFα increased NFκB binding to PGC1α, which downregulated PGC1α and subsequently dysregulated glucose metabolism (Lvarez-Guardia et al., 2010).

### The upsurge of anaerobic glycolytic metabolism

Diabetes causes muscle energy starvation (Simoneau and Kelley, 1997) and DOX causes muscle fatigue (van Norren et al., 2009); hence, it is proposed that when DOX is administered in diabetic skeletal muscle, pro-inflammatory microenvironment upsurge might take place to undergo profound metabolic changes to mediate metabolic adaptation caused by DOX in diabetic skeletal muscle. It has been shown that HIF1α (pro-inflammatory transcription factor) led to upregulation of anaerobic glycolysis and release of lactate (Tannahill et al., 2013). Glycolysis is the metabolic pathway that converts glucose to pyruvate aerobically and lactate anaerobically (Scott, 2008). Interconversion of pyruvate to lactate and vice versa is carried out by LDH during intensive exercise (Spriet, 1995). Consistently, we observed increases in HIF1α and LDH in our examined muscles and it might be possible that the upsurge of pro-inflammatory regulatory molecule has stabilized HIF1α, which led to lactate release posing an aerobic-to-anaerobic metabolic shift. Our interpretation is supported by a study reporting that diabetic skeletal muscle has increased glycolytic rate with upregulation of LDH (Tannahill et al., 2013). The increase in pyruvate dehydrogenase lipoamide kinase isozyme 4 (PDK4) indicated the accumulation of fatty acid, which has been demonstrated to be upregulated in diabetic skeletal muscle (Rosa et al., 2003) and in DOX-treated skeletal muscle (Sin et al., 2015). Nonetheless, HIF1α (pro-inflammatory transcription factor) might also have induced the alteration of PDK4 (Meiser et al., 2016), which was consistent with our findings. Collectively, our results demonstrated that DOX increased the abundances of PDK4, LDH and HIF1α with the downregulation of ACC phosphorylation, and established a pro-inflammatory environment in diabetic skeletal muscle in a HIF1α-dependent manner.

DOX and diabetes individually have been demonstrated to cause the release of ROS that might lead to muscle fatigue and metabolic dysregulation (van Etten et al., 1998; Hardin et al., 2008; van Norren et al., 2009; Ritov et al., 2010), which are in accordance with our present findings of muscle atrophy and insulin signaling markers. Hence, we speculated that DOX in diabetic skeletal muscle might have induced further elevation of ROS production when compared to DOX or diabetes alone. Excessive ROS production has been shown to increase the inflammatory response (Barreiro et al., 2005; Fruehauf and Meyskens, 2007; Zuo et al., 2013a,b) and dysregulate multiple regulatory enzymes such as glutathione peroxidase (GPx), superoxide dismutase (SOD), and catalase (Zuo et al., 2015). In DOX-treated diabetic skeletal muscle, we observed the upregulation of pro-inflammatory microenvironment regulatory molecule (NFkB) and cytokines (TNFα) that might be due to the over-production of ROS (Wang et al., 2011). The balance of ROS has been shown to play an important role in the adaptation and response of the glycolytic activity (Blair et al., 1999). In the present study, the observations on the increase in LDH and PDK4 along with the decrease in ACC leading to dysregulated metabolism suggest that there might be excessive ROS production in DOX-treated diabetic skeletal muscle. High level of glucose or fatty acids is known to enhance ROS production, which plays a vital role in the loss of the number or function of pancreatic beta cells. On the contrary, ROS have been reported to be produced under high or low glucose concentration in pancreatic beta cells by activating AMPK in a superoxide-dependent manner (Sarre et al., 2012). In our results, we found that AMPK was downregulated suggesting that high level of glucose might have resulted in the over-production of ROS in the DOX-treated diabetic skeletal muscle. As ROS play a critical role in inflammation, calcium flux, muscle death and atrophy, diabetes and aerobic metabolism, additional investigation is warranted to comprehensively examine the role of ROS in the modulations of inflammation and metabolism in DOX-treated diabetic muscle.

**Figure 6:**
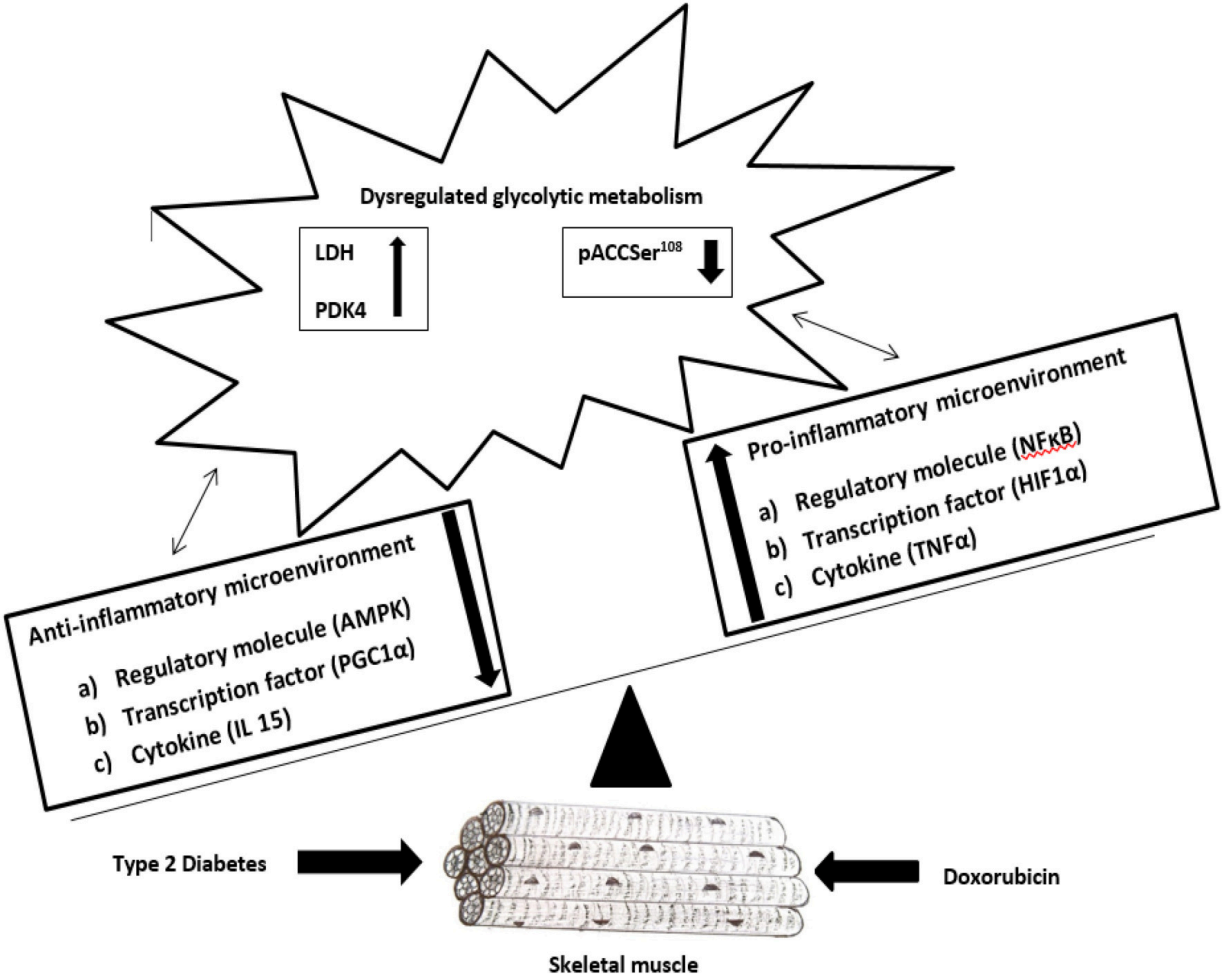
**DOX administration in diabetic skeletal muscle leads to upsurge of pro-inflammatory microenvironment (HIF1α, NFκB, TNFα) along with upsurge of anaerobic glycolytic metabolic regulators (PDK4, LDH) and diminution of anti-inflammatory microenvironment (PGC1α, AMPK, IL15) along with diminution of aerobic metabolic regulator (pACCSer^108^)**.

In conclusion, our data demonstrated that the modulation of the inflammatory pathway might be linked to the shift of oxidative-glycolytic metabolism in diabetic skeletal muscle after DOX exposure (Figure 6). Our findings that the increases in pro-inflammatory transcription factor HIF1α and regulatory molecule NFκB along with the augmentation of inflammatory cytokine TNFα in DOX-injured diabetic muscle might help to reveal the underlying mechanisms and thus hasten the development of new effective strategies to protect skeletal muscle from DOX-induced toxicity in diabetic cancer patients. Moreover, the present findings of the decreases in transcription factor PGC1α and regulatory molecule AMPK along with the diminution of anti-inflammatory myokine IL15 in DOX-injured diabetic muscle might indicate inverse relationship with anti-inflammatory microenvironment and glycolytic metabolism. Although the adopted sample size has resulted in a statistical power of 80% or above for the assessments on muscle mass, IRS1, MAFbx, IL6, IL10, IL15, AMPK, PGC1α, HIF1α, and LDH activity, the constraint of sample size might be a limitation in the present study. Furthermore, additional research is warranted to further investigate the toxic effects of DOX on skeletal muscle in a tumor diabetic animal model.

## Author contributions

RS, BT, XP, and PS designed the studies; RS, BT, and XP performed the experiments; CL, LC, and BY contributed to discussion and editing; RS, BY, and PS analyzed and interpreted data, supervised the project and co-wrote the manuscript.

### Conflict of interest statement

The authors declare that the research was conducted in the absence of any commercial or financial relationships that could be construed as a potential conflict of interest.
